# Association of lifestyle with sleep health in general population in China: a cross-sectional study

**DOI:** 10.1038/s41398-024-03002-x

**Published:** 2024-08-04

**Authors:** Yong-Bo Zheng, Yue-Tong Huang, Yi-Miao Gong, Ming-Zhe Li, Na Zeng, Shui-Lin Wu, Zhi-Bo Zhang, Shan-Shan Tian, Kai Yuan, Xiao-Xing Liu, Michael V. Vitiello, Yu-Mei Wang, Yong-Xiang Wang, Xiu-Jun Zhang, Jie Shi, Le Shi, Wei Yan, Lin Lu, Yan-Ping Bao

**Affiliations:** 1 Peking University Sixth Hospital, Peking University Institute of Mental Health, NHC Key Laboratory of Mental Health (Peking University), National Clinical Research Center for Mental Disorders (Peking University Sixth Hospital), Peking University, Beijing, China; 2 Peking-Tsinghua Centre for Life Sciences and PKU-IDG/McGovern Institute for Brain Research, Peking University, Beijing, China; 3 National Institute on Drug Dependence and Beijing Key Laboratory of Drug Dependence, Peking University, Beijing, China; 4 School of Public Health, Peking University, Beijing, China; 5 Department of Psychiatry and Behavioral Sciences, University of Washington, Seattle, WA USA; 6 Institute of Brain Science and Brain-inspired Research, Shandong First Medical University & Shandong Academy of Medical Sciences, Jinan, Shandong Province 250117 China; 7 School of Psychology, College of Public Health, North China University of Science and Technology, Tangshan, 063210 Hebei Province China; 8 Chinese Academy of Medical Sciences Research Unit (No. 2018RU006), Peking University, Beijing, China; 9 Shandong Institute of Brain Science and Brain-inspired Research; Medical Science and Technology Innovation Center, Shandong First Medical University & Shandong Academy of Medical Sciences, Jinan, Shandong Province 271016 China

**Keywords:** Psychiatric disorders, Scientific community

## Abstract

The concept of a healthy lifestyle is receiving increasing attention. This study sought to identify an optimal healthy lifestyle profile associated with sleep health in general population of China. An online cross-sectional survey was conducted from June to July 2022. Six healthy lifestyle factors were assessed: healthy diet, regular physical exercise, never smoking, never drinking alcohol, low sedentary behavior, and normal weight. Participants were categorized into the healthy lifestyle (5-6 factors), average (3-4 factors), and unhealthy lifestyle groups (0-2 factors). The study’s primary outcome was sleep health, which included sleep quality, duration, pattern, and the presence of any sleep disorder or disturbance, including insomnia, excessive daytime sleepiness, obstructive apnea syndrome, and narcolepsy. Multivariable logistic regression analysis was applied to explore lifestyles associated with the selected sleep health outcomes. 41,061 individuals were included, forming 18.8% healthy, 63.8% average, and 17.4% unhealthy lifestyle groups. After adjusting for covariates, participants with healthy lifestyle were associated with a higher likelihood of good sleep quality (OR = 1.56, 95% CI = 1.46–1.68), normal sleep duration (OR = 1.60, 95% CI = 1.49–1.72), healthy sleep pattern (OR = 2.15, 95% CI = 2.00–2.31), and lower risks of insomnia (OR = 0.66, 95% CI = 0.61–0.71), excessive daytime sleepiness (OR = 0.66, 95% CI = 0.60–0.73), and obstructive apnea syndrome (OR = 0.40, 95% CI = 0.37–0.43), but not narcolepsy (OR = 0.92, 95% CI = 0.83–1.03), compared to those with unhealthy lifestyle. This large cross-sectional study is the first to our knowledge to quantify the associations of a healthy lifestyle with specific aspects of sleep health. The findings offer support for efforts to improve sleep health by modulating lifestyle.

## Introduction

Sleep is a core behavior which consumes a third of every day. Sleep health is generally regarded as a vital health condition that influences an individuals’ happiness, quality of life, and sense of well-being and is also associated with physical and psychological health [1, 2]. Sleep health is a complex concept consisting of sleep quality, duration, pattern, and sleep disorders or disturbances (e.g., insomnia, excessive daytime sleepiness [EDS], obstructive sleep apnea syndrome [OSAS], and narcolepsy, etc.) [1, 2]. Impaired sleep health has been demonstrated to increase risk of mortality, cardiovascular events, and psychiatric disorders [3, 4]. Conversely, improving sleep health, and preventing sleep disorders can help to ensure overall physical and mental wellbeing. Fortunately, the various components of sleep health can be improved through a variety of empirically supported interventions [5].

Numerous studies have explored factors that may influence sleep health, including genotype, age, and lifestyle. Among these, lifestyle has received increasing attention; particularly because aspects of lifestyle can be improved with potential benefits for overall health, including mortality [6], cardiovascular diseases [7], and cognitive decline [8]. As for the relationship between aspects of lifestyle and sleep health, studies have explored the association of healthy lifestyle with OSAS, restless legs syndrome, and non-restorative sleep [9–11]. The majority of these studies have focused on the impact of a single component of a healthy lifestyle, including diet [12], exercise [13], smoking [14], alcohol drinking [15], sedentary behavior [16], or body weight [17] on sleep health outcomes. Identification and modification of lifestyle factors associated with aspects of sleep health may improve sleep health as well as physical and mental wellbeing. Such studies may also inform investigation of sleep disorder pathophysiology.

Understanding how lifestyle factors are associated with sleep health is a key component of building accurate lifestyle education into routine clinical practice. However, there is still a lack of evidence demonstrating the associations of a healthy lifestyle and its components with sleep health and its components and studies examining these associations are needed to address this knowledge gap. Here we conduct an online survey among the general population from 34 provinces in China, aiming to (1) investigate the pattern and characteristics distribution of lifestyle and sleep health in general population in China, and (2) further to explore the association of healthy lifestyle factors and the sleep health with various components, including sleep quality, duration, pattern, disturbances, and disorders. Study finding will help us better understand the nature of sleep health pattern in a large sample of Chinese participants and provide support for the promotion of sleep health to improve quality of life and psychical and mental wellbeing.

## Methods

### Study design, participants, and data collection

This cross-sectional online survey drawn from the general population of 34 provinces, municipalities and autonomous regions of China was conducted from June 12^th^ to July 17^th^, 2022. A self-designed questionnaire was released via the health page in the Chinese website Joybuy, a large e-commerce with 580 million users and information service platform that provides online health products and services in China [18, 19]. The survey link was posted on the website to all users without advertisement or commercial interest. A convenience sampling method was used in this study. The registered members of this online health platform clicked the survey link on the website and responded to the survey voluntarily [18, 19]. Informed consent was received online before the respondents began the questionnaire. Participants who completed all necessary questions could submit the questionnaire and would receive small amount shopping vouchers after submission. Participation was anonymous and all information was de-identified. The study was conducted in compliance with ethical standards depicted in the Helsinki declaration and approved by the ethics committee of Peking University Sixth Hospital (protocol code 2022-6-10-1 and date of approval: 10 June 2022). All participants provided informed consent before the respondents began the questionnaire.

**Fig. 1:**
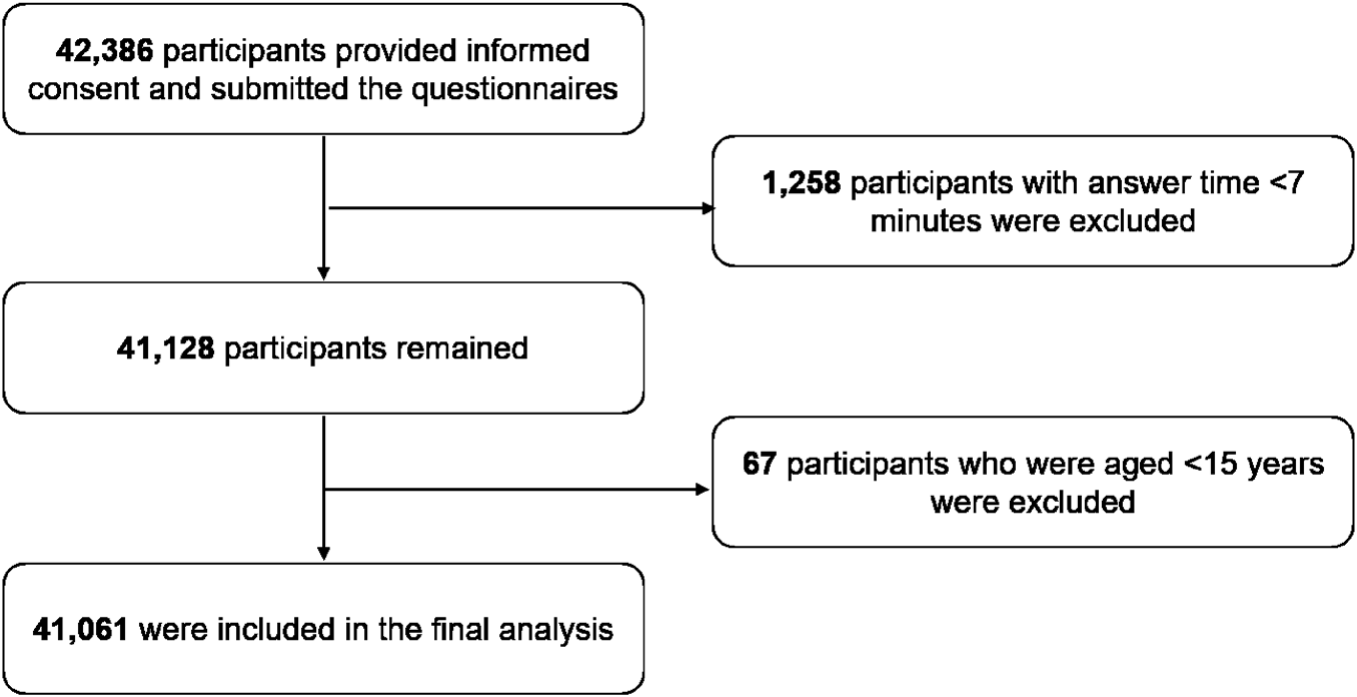
Flow chart of participants recruitment.

A total of 42,386 individuals provided informed consent and submitted questionnaires. 1258 participants who had answer times less than 7 minutes were excluded (previous testing had shown that the questionnaires could not be validly completed in such a brief time), and 67 respondents younger than 15 years of age were excluded, leaving 41,061 participants (96.9%) for analysis. Details of the recruitment process are presented in Fig. 1.

### Classification of healthy lifestyle categories

We calculated a healthy lifestyle score for all participants using six components: diet, physical exercise, smoking, drinking alcohol, sedentary behavior, and body weight, based on the literature [8, 20]. For diet, we recorded the participant’s daily intake of 7 healthy food items (pork, fish, eggs, vegetables, legumes, fruits, dairy products) and 2 unhealthy food items (sweeties, oiled food) [21]. A healthy diet was defined as frequently taking at least 4 of 7 healthy foods (≥ 4 days a week), and seldom taking above two unhealthy food (≤3 days a week). For physical exercise, weekly frequency, duration, and vigorousness were collected. At least 150 min of mild or 75 min of moderate-to-vigorous activity per week was considered healthy, based on guidelines, and established literature [20, 22]. For smoking, participants who never smokes were recognized as a healthy lifestyle factor [23]. Similarly, participants who never drank were deemed as a healthy lifestyle factor [24]. Sedentary behavior less than 6 hours a day [25] and normal weight (BMI within 18.5–24.9) [26] were each defined as healthy lifestyle factors. Detailed questions related to a healthy lifestyle are listed in Supplementary Table 1.

For each factor, healthy was assigned 1 point and otherwise 0 point. The healthy lifestyle score was the sum of all six factors, ranging from 0 to 6, with a higher score indicating better adherence to an overall healthy lifestyle. Participants were categorized into the healthy lifestyle group if they had a score of 5-6), into the average group if they had a score of 3–4), and into the unhealthy lifestyle group if they had a score of 0–2.

### Assessment of sleep health

Four components of sleep health were assessed: sleep quality, duration, pattern, and the presence of any sleep disorder or disturbance (e.g., insomnia, EDS, OSAS, and narcolepsy) and served as the study outcome measures [1, 2].

Sleep quality was assessed using the Pittsburgh Sleep Quality Index (PSQI), a questionnaire consisting of 19 items to assess sleep quality in the past month [27]. PSQI scores ranged from 0 to 21, with scores less than 7 as good sleep quality [28].

Sleep duration was categorized as short sleep (≤6 hours/night), normal sleep (7-9 hours/night) and long sleep (≥ 10 hours/night) [29].

We integrated five sleep traits (chronotype, sleep duration, insomnia, snoring, and daytime sleepiness) to generate a healthy sleep pattern score, as previously described [30, 31]. Healthy sleep traits were defined as follows: early chronotype (‘definite morning’ or ‘morning than evening’); 7–8 h sleep duration; never or rarely having insomnia symptoms; no snoring; and no frequent daytime sleepiness (‘never/rarely’ or ‘sometimes’). If participants reported a healthy sleep trait, they got a score of 1; otherwise, they received 0. All five sleep traits scores were added up to obtain a sleep pattern score. The sleep pattern score ranged from 0 to 5, with higher scores indicating a healthier sleep pattern. We then classified scores of 4 or 5 as healthy, scores of 2 or 3 as intermediate and 0 or 1 as poor sleep pattern scores.

The presence or absence of sleep disorders or disturbances, including insomnia, EDS, OSAS, and narcolepsy, were assessed using Insomnia Screening Index (ISI), Epworth Sleepiness Scale (ESS), Stop-Bang questionnaire (SBQ), and Ullanlinna Narcolepsy Scale (UNS). The ISI is a 7-item self-report questionnaire assessing the nature, severity, and impact of insomnia. The total score of ISI ranged from 0 to 28, with score equal to or over 8 indicating sub-threshold insomnia [32]. The ESS is a self-administered questionnaire, comprising 8 questions that survey the likelihood of daytime sleepiness. The total score of ESS ranged from 0 to 24, with a score equal to or over 11 indicating EDS [33]. SBQ was specifically developed as a reliable screening tool for OSAS, with a score equal to or over 3 indicating potential OSAS [34]. UNS is a simple questionnaire-based method which was used to measure the symptoms of the narcoleptic syndrome, with scores equal to or over 14 indicating narcolepsy symptom [35].

### Covariates

Demographic characteristics (e.g., age, sex, ethnicity, educational attainment, income level, marital status, type of jobs, living area, and living alone), sleeping habit (e.g., napping habit, and shift work), medical comorbidities (e.g., history of chronic disease, mental illness, depression symptoms, and anxiety symptoms), and COVID-19 status (e.g., infection status of participants, and experiences of quarantine during COVID-19 epidemics) were collected via questionnaire. Detailed questions related to demographic information are reported in Supplementary Table 1.

Depression and anxiety symptoms were assessed using Patient Health Questionnaire–2 [36], and Generalized Anxiety Disorder–2 [37]. Both scales use a cut-off of greater than 3 for the presence of depression and of anxiety symptoms [36–38].

### Statistical analysis

Descriptive statistics were used to present demographic characteristics, sleeping habits, medical comorbidities, and information related to COVID-19 in total sample, as well as healthy, average, and unhealthy lifestyle groups. In addition, proportions of healthy lifestyle, and sleep health outcomes stratified by three healthy lifestyle groups were also described. Continuous variables were presented as mean (SD) for normal distribution variables or median (IQR) for non-normal distribution. Binary and categorical variables were presented as counts and percentages. χ2 tests and one-way analysis of variance (ANOVA) were used to compare categorial and continuous variables among three groups, and related *p*-values are presented in Table 1.

Multivariable logistic regression analysis was performed to calculate the odds ratios (ORs) and 95% confidential intervals (CI) of association of lifestyle and sleep health outcomes (i.e., sleep quality, duration, pattern, and disorders or disturbances), after adjusting covariates, including demographic characteristics, sleeping habit, medical comorbidities, and information related to COVID-19. Similarly, logistic regression analysis examined the associations of each health lifestyle component with sleep health outcomes, after adjusting for the covariates mentioned above. The level of significance was set to *P* < 0.05. All the statistical analyses were performed using SPSS statistical software version 22 (IBM Corp).

## Results

### Demographic characteristics

In this cross-sectional, nationwide online survey, a convenience sample totaling 41,061 participants were included in the final analysis. Participants included in the analysis represented all thirty-four province-level regions in China. The participants’ average age was 36.24 ± 9.51 years, and the majority were female (59.1%), of Han ethnicity (96.6%), college educated (72.8%), married (74.3%), involved in non-manual work (65.8%), living in urban area (91.7%), and not living alone (83.6%). In addition, 45.5% of participants were frequent nappers, and 23.4% of them were shift workers. 17.3% reported chronic medical conditions, and 2.3% reported chronic mental illness. 19.4% reported depression symptoms and 16.7% reported anxiety symptoms. Regarding COVID-19 status, few participants were currently infected (1.7%), and few of them had quarantine history (6.4%) or were currently quarantined (0.9%). Additional demographic characteristics, sleeping habits, medical comorbidities, and information related to COVID-19 of participants stratified by healthy lifestyle are reported in Table 1, with all *p*-values < 0.05.

**Table 1:** Demographic characteristics of the total sample and stratified by healthy lifestyle.

	Total sample (*N* = 41061)	Unhealthy lifestyle group (*N* = 7715)	Average group (*N* = 26186)	Healthy lifestyle group (*N* = 7160)	*p*-value
**Age (mean** **±** **SD)**	36.24 ± 9.51	36.27 ± 9.37	35.71 ± 9.28	38.14 ± 10.24	< 0.001
**Sex**					< 0.001
Male	16814 (40.9)	4489 (58.2)	9905 (37.8)	2420 (33.8)	
Female	24247 (59.1)	3226 (41.8)	16281 (62.2)	4740 (66.2)	
**Ethnicity**					0.027
Han	39675 (96.6)	7418 (96.2)	25320 (96.7)	6937 (96.9)	
Others	1386 (3.4)	297 (3.8)	866 (3.3)	223 (3.1)	
**Education attainment**					< 0.001
High school or lower	7784 (19.0)	1706 (22.1)	4806 (18.4)	1272 (17.8)	
College or university	29874 (72.8)	5449 (70.6)	19187 (73.3)	5238 (73.2)	
Postgraduate	3403 (8.3)	560 (7.3)	2193 (8.4)	650 (9.1)	
**Income level (yuan/month)**					< 0.001
≤ 4999	12279 (29.9)	2289 (29.7)	7976 (30.5)	2014 (28.1)	
5000–19999	22084 (53.8)	4184 (54.2)	14079 (53.8)	3821 (53.4)	
≥20000	6698 (16.3)	1242 (16.1)	4131 (15.8)	1325 (18.5)	
**Marital status**					< 0.001
Unmarried	9579 (23.3)	1908 (24.7)	6371 (24.3)	1300 (18.2)	
Married	30514 (74.3)	5587 (72.4)	19224 (73.4)	5703 (79.7)	
Separate, widow, or others	968 (2.4)	220 (2.9)	591 (2.3)	157 (2.2)	
**Type of jobs**^**a**^					< 0.001
Non-manual work	27020 (65.8)	5146 (66.7)	17416 (66.5)	4458 (62.3)	
Manual work	7394 (18.0)	1570 (20.3)	4603 (17.6)	1221 (17.1)	
Jobless or retired	6647 (16.2)	999 (12.9)	4167 (15.9)	1481 (20.7)	
**Living area**					< 0.001
Rural	3398 (8.3)	704 (9.1)	2237 (8.5)	457 (6.4)	
Urban	37663 (91.7)	7011 (90.9)	23949 (91.5)	6703 (93.6)	
**Living alone**					< 0.001
Yes	6728 (16.4)	1507 (19.5)	4266 (16.3)	955 (13.3)	
No	34333 (83.6)	6208 (80.5)	21920 (83.7)	6205 (86.7)	
**Napping habit**^**b**^					< 0.001
No	8118 (19.8)	1684 (21.8)	5258 (20.1)	1176 (16.4)	
Less frequently	14255 (34.7)	2795 (36.2)	9138 (34.9)	2322 (32.4)	
Frequently	18688 (45.5)	3236 (41.9)	11790 (45.0)	3662 (51.1)	
**Shift work**					< 0.001
Yes	9612 (23.4)	2472 (32)	5823 (22.2)	1317 (18.4)	
No	31449 (76.6)	5243 (68)	20363 (77.8)	5843 (81.6)	
**History of chronic disease**					< 0.001
Yes	7114 (17.3)	1777 (23.0)	4255 (16.2)	1082 (15.1)	
No	33947 (82.7)	5938 (77.0)	21931 (83.8)	6078 (84.9)	
**History of mental illness**					< 0.001
Yes	926 (2.3)	244 (3.2)	570 (2.2)	112 (1.6)	
No	40135 (97.7)	7471 (96.8)	25616 (97.8)	7048 (98.4)	
**Depression symptoms**^**c**^					< 0.001
Yes	7959 (19.4)	1951 (25.3)	5114 (19.5)	894 (12.5)	
No	33102 (80.6)	5764 (74.7)	21072 (80.5)	6266 (87.5)	
**Anxiety symptoms**^**c**^					< 0.001
Yes	6867 (16.7)	1713 (22.2)	4368 (16.7)	786 (11.0)	
No	34194 (83.3)	6002 (77.8)	21818 (83.3)	6374 (89.0)	
**Have you ever been infected with COVID-19?**					< 0.001
No	39576 (96.4)	7323 (94.9)	25278 (96.5)	6975 (97.4)	
Asymptomatic infection	807 (2.0)	230 (3.0)	484 (1.8)	93 (1.3)	
Confirmed infection	678 (1.7)	162 (2.1)	424 (1.6)	92 (1.3)	
**Have you ever been quarantined during COVID-19 epidemics?**					< 0.001
No	38036 (92.6)	7030 (91.1)	24278 (92.7)	6728 (94.0)	
Ever quarantined	2641 (6.4)	575 (7.5)	1666 (6.4)	400 (5.6)	
Being quarantined currently	384 (0.9)	110 (1.4)	242 (0.9)	32 (0.4)	

*COVID-19* Coronavirus disease 2019.

^a^Non-manual work included civil servants, institutional staff, enterprise or company employees, freelancers (e.g., writers, artists, anchors, etc.), medical workers; manual work included private or individual workers, service workers, servicemen or military occupations, workers, and farmers; jobless or retired included students, full-time caretaker of a family, unemployed or jobless, and retired.

^b^Napping occasionally (1–3 times/month) and sometimes (1-2 times/week) were defined as less frequently napping habit; Napping often (3–5 times/week) and almost every day were defined as frequently napping habit.

^c^Patient Health Questionnaire–2 score ≥ 3 indicated depression symptoms; and Generalized Anxiety Disorder≥3 indicated anxiety symptoms.

### Proportion of sleep health outcomes in healthy, average, and unhealthy lifestyle groups

**Fig. 2:**
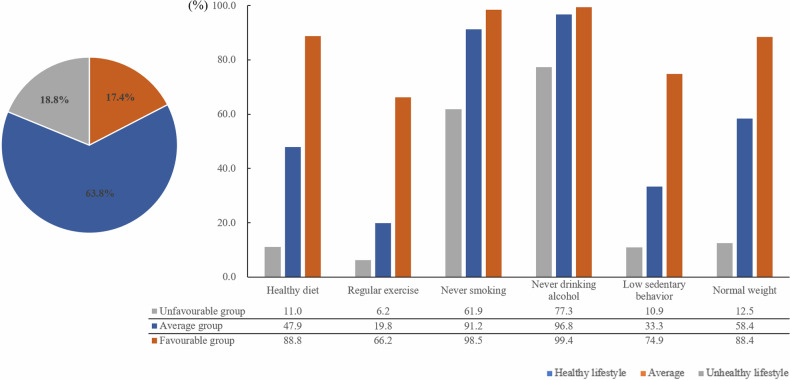
Proportions of each healthy lifestyle component in healthy, average, and unhealthy lifestyle groups.

For the entire sample, the proportion of participants practicing each healthy lifestyle component was 48.1% healthy diet, 25.3% regular physical exercise, 87.6% never smoked, 93.6% never drank alcohol, 36.4% low sedentary behavior, and 55.0% normal weight (Supplementary Fig. 1). Figure 2a presents the proportions of participants stratified by lifestyle; 18.8% healthy lifestyle, 63.8% average lifestyle, and 17.4% unhealthy lifestyle. Participants in the healthy lifestyle group, by definition, reported higher proportions of all healthy lifestyle components when compared to the average and unhealthy lifestyle groups; healthy diet (88.8% vs. 47.9% vs. 11.0%; *p* < 0.001), regular physical exercise (66.2% vs. 19.8% vs. 6.2%; *p* < 0.001), never smoked (98.5% vs. 91.2% vs. 61.9%; *p* < 0.001), never drank alcohol (99.4% vs. 96.8% vs. 77.3%; *p* < 0.001), low sedentary behavior (74.9% vs. 33.3% vs. 10.9%; *p* < 0.001), and normal weight (88.4% vs. 58.4% vs. 12.5%; *p* < 0.001). Proportions of each healthy lifestyle component in healthy, average, and unhealthy lifestyle groups are presented in Fig. 2b.

**Fig. 3:**
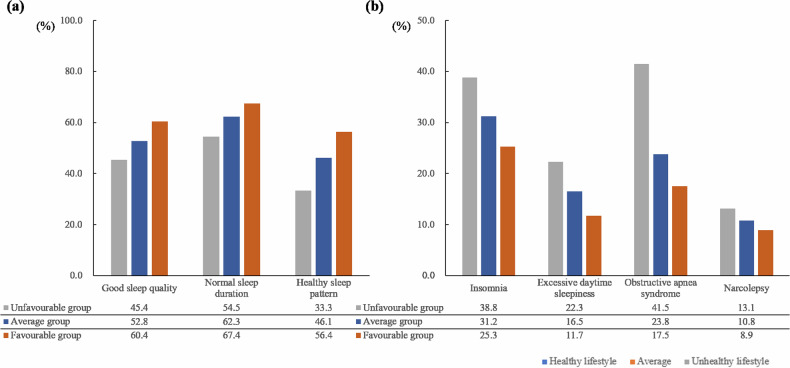
Proportions of sleep health outcomes in different lifestyle groups. **a** Proportions of good sleep quality, normal sleep duration, and healthy sleep pattern in healthy, average, and unhealthy lifestyle groups. **b** Proportions of insomnia, excessive daytime sleepiness, obstructive apnea syndrome, and narcolepsy in healthy, average, and unhealthy lifestyle groups.

For the entire sample, 52.8% reported good sleep quality, 58.6% normal sleep duration, and 45.5% healthy sleep pattern. The sample proportions for sleep disorders or disturbances were 31.6% insomnia, 16.8% EDS, 26.1% OSAS, and 10.9% narcolepsy (Supplementary Fig. 2). Figure 3 presents the proportions of sleep health outcomes by lifestyle. The proportions of sleep health components in the healthy lifestyle group were significantly higher compared to the average and unhealthy lifestyle groups: good sleep quality (60.4% vs. 52.8% vs. 45.4%; *p* < 0.001), normal sleep duration (64.2% vs. 59.1% vs. 51.4%; *p* < 0.001), and healthy sleep pattern (56.4% vs. 46.1% vs. 33.3%; *p* < 0.001); while the proportions of insomnia (25.3% vs. 31.2% vs. 38.8%; *p* < 0.001), EDS (11.7% vs. 16.5% vs. 22.3%; *p* < 0.001), OSA (17.5% vs. 23.8% vs. 41.5%; *p* < 0.001), and narcolepsy (8.9% vs. 10.8% vs. 13.1%; *p* < 0.001) were significantly lower.

### Association of healthy lifestyle with sleep health

**Fig. 4:**
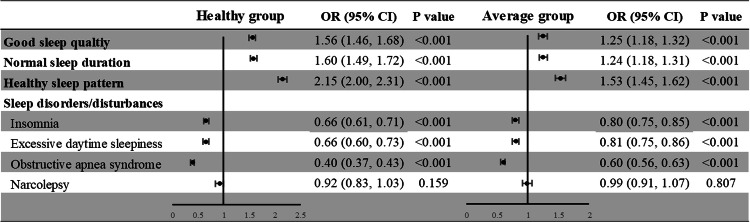
Forest plots of results of odds ratios of sleep health outcome in healthy and average lifestyle groups, compared with participants in the unhealthy lifestyle group.

Figure 4 presents the association of healthy lifestyle and sleep health among the participants. Participants in the healthy lifestyle group compared to those reporting unhealthy lifestyle were more likely to have good sleep quality (OR = 1.56, 95% CI = 1.46-1.68, *p* < 0.001), normal sleep duration (OR = 1.60, 95% CI = 1.49–1.72, *p* < 0.001), a healthy sleep pattern (OR = 2.15, 95% CI = 2.00-2.31, *p* < 0.001), and lower risk of insomnia (OR = 0.66, 95% CI = 0.61-0.71, *p* < 0.001), EDS (OR = 0.66, 95% CI = 0.60–0.73, *p* < 0.001), and OSAS (OR = 0.40, 95% CI = 0.37-0.43, *p* < 0.001), but not narcolepsy (OR = 0.92, 95% CI = 0.83-1.03, *p* = 0.159) after adjusting covariates.

Similarly, participants in the average compared to the unhealthy lifestyle group had higher ratio for good sleep quality (OR = 1.25, 95% CI = 1.18–1.32, *p* < 0.001), normal sleep duration (OR = 1.24, 95% CI = 1.18–1.31, *p* < 0.001), healthy sleep pattern (OR = 1.53, 95% CI = 1.45–1.62, *p* < 0.001), and low ORs for sleep disorders or disturbances, including insomnia (OR = 0.80, 95% CI = 0.75–0.85, *p* < 0.001), EDS (OR = 0.81, 95% CI = 0.75–0.86, *p* < 0.001), and OSAS (OR = 0.60, 95% CI = 0.56–0.63, *p* < 0.001), but not narcolepsy (OR = 0.99, 95% CI = 0.91–1.07, *p* = 0.807).

In addition, participants reporting shift work, a history of chronic diseases, depression symptoms, or anxiety symptoms were each associated with all sleep health outcomes, including lower likelihood of good sleep quality, normal sleep duration, and a healthy sleep pattern, as well as higher risk of all sleep disorders or disturbances including insomnia, EDS, OSAS, and narcolepsy symptoms (Supplementary Table 2 and 3).

### Association of each healthy lifestyle component with sleep health

**Fig. 5:**
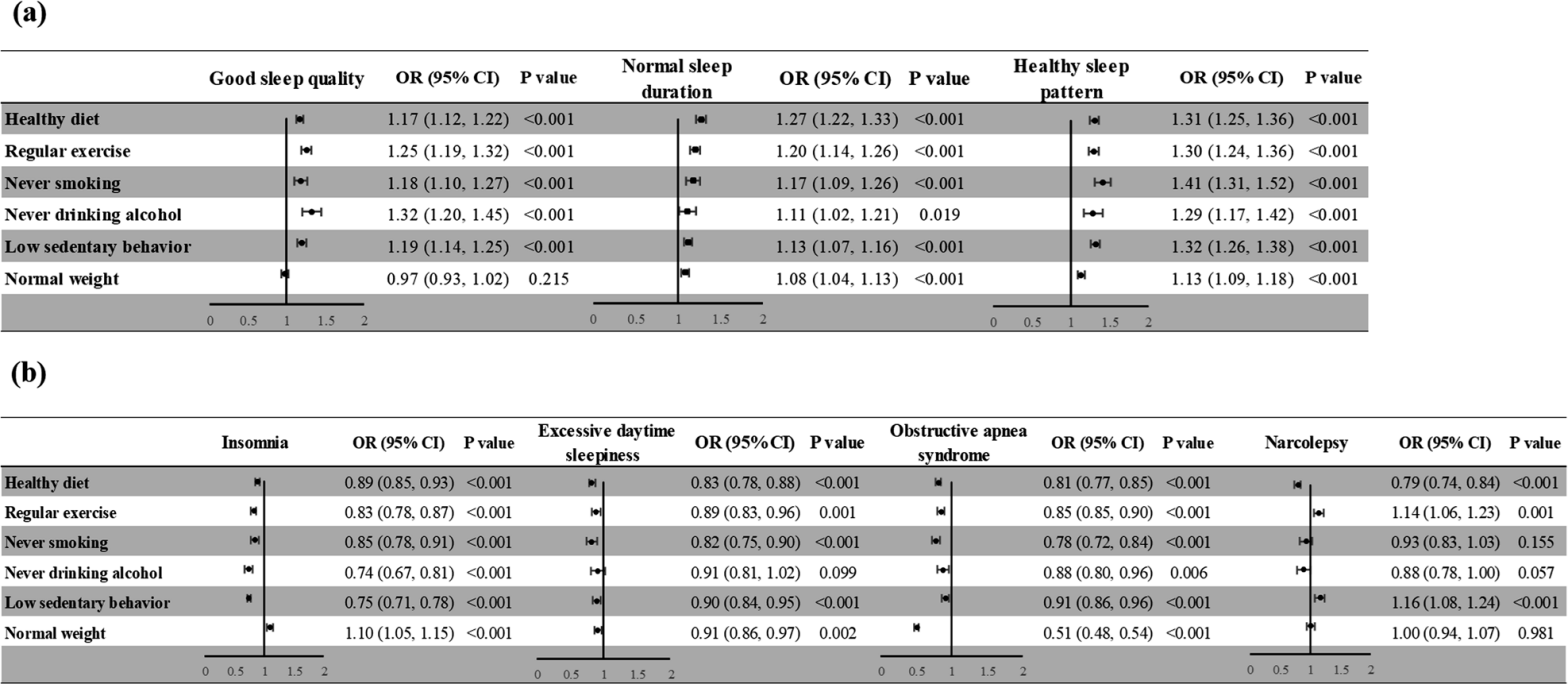
Forest plots of results of odds ratios of each healthy lifestyle component and sleep health outcomes. **a** Results of odds ratios of each healthy lifestyle component with good sleep quality, normal sleep duration, and healthy sleep pattern. **b** Results of odds ratios of each healthy lifestyle component with insomnia, excessive daytime sleepiness, obstructive apnea syndrome, and narcolepsy.

Figure 5 presents the association of each healthy lifestyle component and sleep health among the participants. Overall, participants with healthy diet, regular exercise, never smoking, and low sedentary behavior were associated with a higher likelihood of good sleep quality, normal sleep duration, healthy sleep pattern, and lower risks of insomnia, EDS, and OSA, with all p value < 0.001; participants with never drinking was associated with above health outcomes with the exception of OSA; participants with normal weight were associated with a higher likelihood of normal sleep duration, healthy sleep pattern, and insomnia, and lower likelihood of EDS and OSA. For narcolepsy, participants with healthy diet exhibited lower risk, while those with regular exercise and low sedentary behavior exhibited higher risks. Detailed results are presented in Supplementary Tables 4 and 5.

## Discussion

This large cross-sectional study is the first to our knowledge to quantify the comprehensive associations of a healthy lifestyle with various aspects of sleep health. Our results showed that a healthy lifestyle was associated with a higher likelihood of good sleep quality, normal sleep duration, a healthy sleep pattern, and fewer sleep disorders, including insomnia, EDS, and OSAS, but not narcolepsy. The findings inform efforts to improve sleep health by modulating healthy lifestyle behaviors.

Lifestyle and its relationship to sleep is being studied with growing interest. Previous studies have reported that aspects of a healthy lifestyle were associated with lower risks for OSA [10], restless legs syndrome [9], and non-restorative sleep [11]. These studies have typically focused on a single lifestyle factor, such as smoking, drinking, diet, or physical activities and their relationship to sleep health [12–15], However, to the best of our knowledge no study has been conducted to examine the relationship between multiple healthy lifestyle behaviors and multiple sleep health outcomes. In this study, we found that individuals who reported engaging in multiple healthy lifestyle behaviors experienced a significantly higher likelihood of good sleep quality, normal sleep duration, and healthy sleep pattern, as well as fewer sleep disorders or disturbances, comparing with those reporting fewer such behaviors. These findings support the potential positive association of healthy lifestyle and sleep health and suggest that increasing healthy lifestyle behaviors may improve sleep health.

Although the mechanism responsible for such changes was not determined in this study, we propose the following potential explanations: 1) Regulation of circadian rhythm: Healthy lifestyle, especially regular exercise and healthy diet, seems to be able to reset disruptions in circadian pacemakers and compensate dysregulation of sleep homeostasis [39, 40]. 2) Inhibition of oxidative stress and inflammation: Oxidative stress, increase in pro-inflammatory markers, imbalance in NO production, and endothelium impairment have been suggested to be correlated with sleep disorders [41, 42]. Reduced smoking and drinking, low sedentary behaviors, and abundant exercise all have been implied to inhibit oxidative stress and inflammation [8]. 3) Regulation of neurohormones and neurotrophic factors: Promotion of neurotrophic factors [43], such as brain-derived neurotrophic factor, plays an important role in sleep disorders; in the meantime, neurohormones such as pregnanolone, allopregnanolone and pregnenolone are involved in the generation of slow wave sleep [44]. Lifestyle has been suggested influence these neurohormones and neurotrophic factors [8]. 4) Involvement of microbiota-gut-brain axis: Lifestyle plays a role in microbiota-gut-brain axis [45], which may potentially affect sleep health [46]. Any one or each of these can potentially contribute to the impact of particular lifestyle behaviors on particular aspects of sleep health. A fuller understanding of how a healthy lifestyle may improve sleep health outcomes remains to be explored.

Consistent with previous evidence [3, 12–14, 16], healthy diet, regular exercise, never smoking, and low sedentary behavior were significantly associated with good sleep quality, normal sleep duration, healthy sleep pattern, and fewer sleep disorders or disturbances, suggesting these factors are robust in regulating sleep health. However, it was not our expectation that normal weight had higher risk for insomnia. A meta-analysis suggested association between weight and insomnia were inconclusive [47]. There may be other covariates confound this association. Further research is needed to address this issue.

It is of note that many of the sleep health outcomes examined in this study can potentially be modulated by modifiable lifestyle factors, with the apparent exception of narcolepsy. The exact cause of narcolepsy is unknown although there are several possibilities. However, more than 98% of people with type 1 narcolepsy carry HLA-DQB1*06:02, indicating that genetics plays a predominant role in narcolepsy [48, 49]. As a highly genetically determined disease, narcolepsy may not be easy to be modified by combined healthy behaviors. To date, no daily lifestyle has been mentioned to hinder or slow disease development of narcolepsy [49, 50]. Different from narcolepsy, other sleep health outcomes are likely to be amenable to improvement in response to healthy lifestyle behaviors. Compared with individuals reporting a healthy lifestyle, those reporting an unhealthy lifestyle had a 2-fold increased risk of OSAS in the current study and weight loss and exercise are essential in the treatment of OSAS [51]. Other studies have reported the roles of healthy diet, exercise, as well as avoiding smoking in the prevention of insomnia [52, 53]. Overall, our findings suggest the potential to modulate many sleep health outcomes by adopting a healthier lifestyle.

We also identified other modifiable factors, including shift work, history of chronic diseases, depression symptoms, and anxiety symptoms that were all negatively associated with each of the sleep health outcomes. The adverse relationship of shift work to multiple health outcomes mimic those of insufficient sleep; unfortunately, it is difficult to intervene in the sleep problems caused by shift work [54]. While the relationship between chronic diseases, depression, and anxiety symptoms with sleep health outcomes were generally bidirectional [55], and it is hard to tell cause-and-effect; nevertheless, factors including chronic diseases, depression, and anxiety symptoms are treatable, which may beneficially influence sleep health [56].

### Strengths and limitations

To our knowledge, this cross-sectional study with a large sample of the general Chinese population is the first study to provide an opportunity to comprehensively observe the association of lifestyle profiles and sleep health. However, our study has limitations. First, this was an online survey, in which convenience sampling was used. Although this study had extensive geographic coverage across China and a large sample size, it was conducted among internet users who were young, highly educated, and from urban areas. Therefore, the representativeness of the sample is limited, particularly for older adults from rural settings. In the meantime, the proportion of individuals with an unhealthy lifestyle might have been underestimated as people with poor health were less likely to participate in the study [8]. Second, the assessments of lifestyle factors (e.g., diet, physical exercise, and alcohol drinking, etc.) were based on self-reported questions, but not validated instruments, which might be prone to measurement error and recall bias. Third, some of the components of healthy lifestyle were based on expert knowledge and data distribution of the included sample, as some questions raised in the self-designed questionnaire were different from established guidelines. Fourth, the study employed a cross-sectional design, which limits its findings to associations and does not allow examination of causal relationships.

## Conclusions

This large cross-sectional study is the first to our knowledge to estimate the association of different lifestyle profiles and sleep health in the general population. The results showed a consistent and significant association of healthy lifestyle and sleep health. Study findings suggest the potential to modulate many sleep health outcomes by adopting a healthy lifestyle and may be useful in guiding individual lifestyle choice as well as clinical practice and public policy.

### Supplementary information

Supplemental Material

## Data Availability

The data and statistical analysis plan in this study are available for others after publication. To access this data, please email one of our corresponding authors.
