# Supplementary material for: Association of lifestyle with sleep health in general population in China: a cross-sectional study
Source: Transl Psychiatry. 2024 Aug 4;14:320. doi: 10.1038/s41398-024-03002-x (PMC11298538; doi:10.1038/s41398-024-03002-x)
Supplement: Supplementary file 1 — Supplemental Material [file 41398_2024_3002_MOESM1_ESM.docx]

**Supplementary Fig. 1. Proportions of each healthy lifestyle component in general population in China.**

**
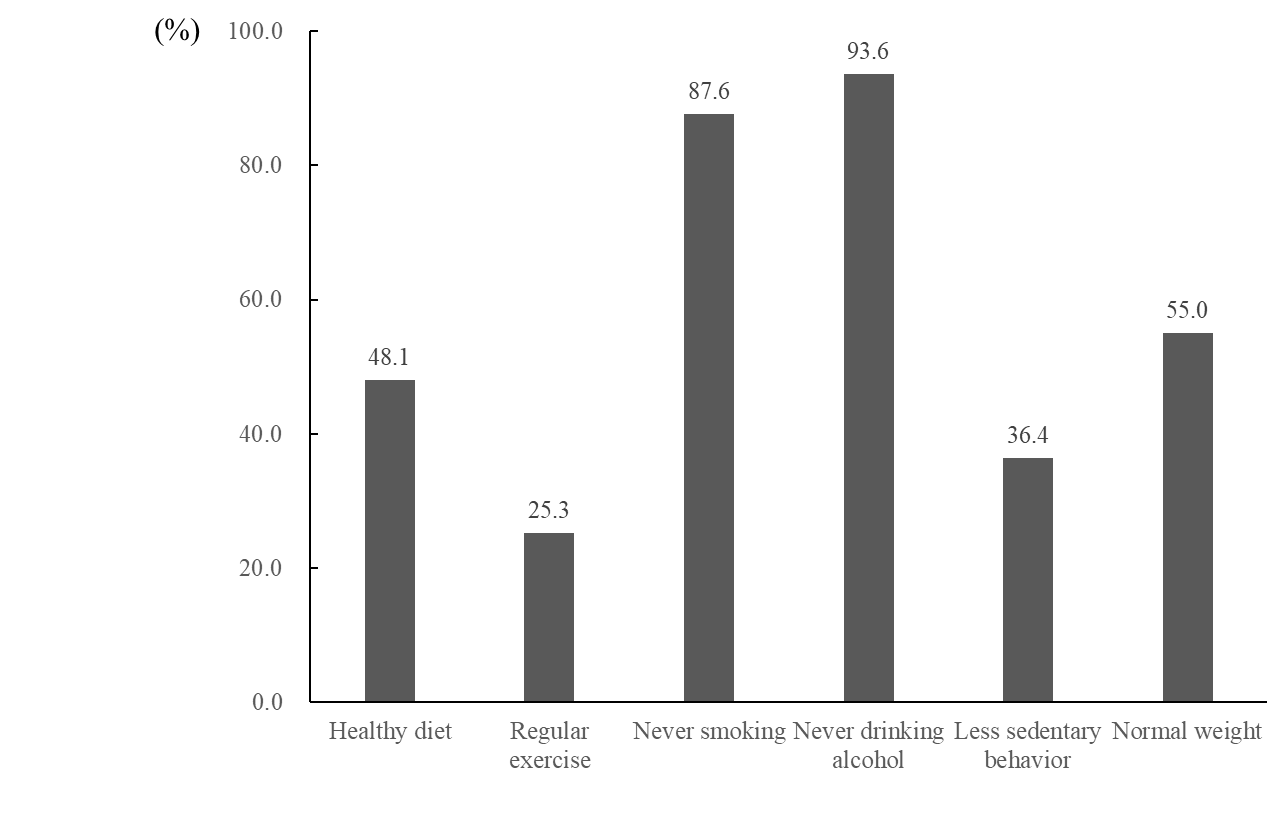
**

**Supplementary Fig. 2. Proportions of sleep health outcomes in general population in China.**


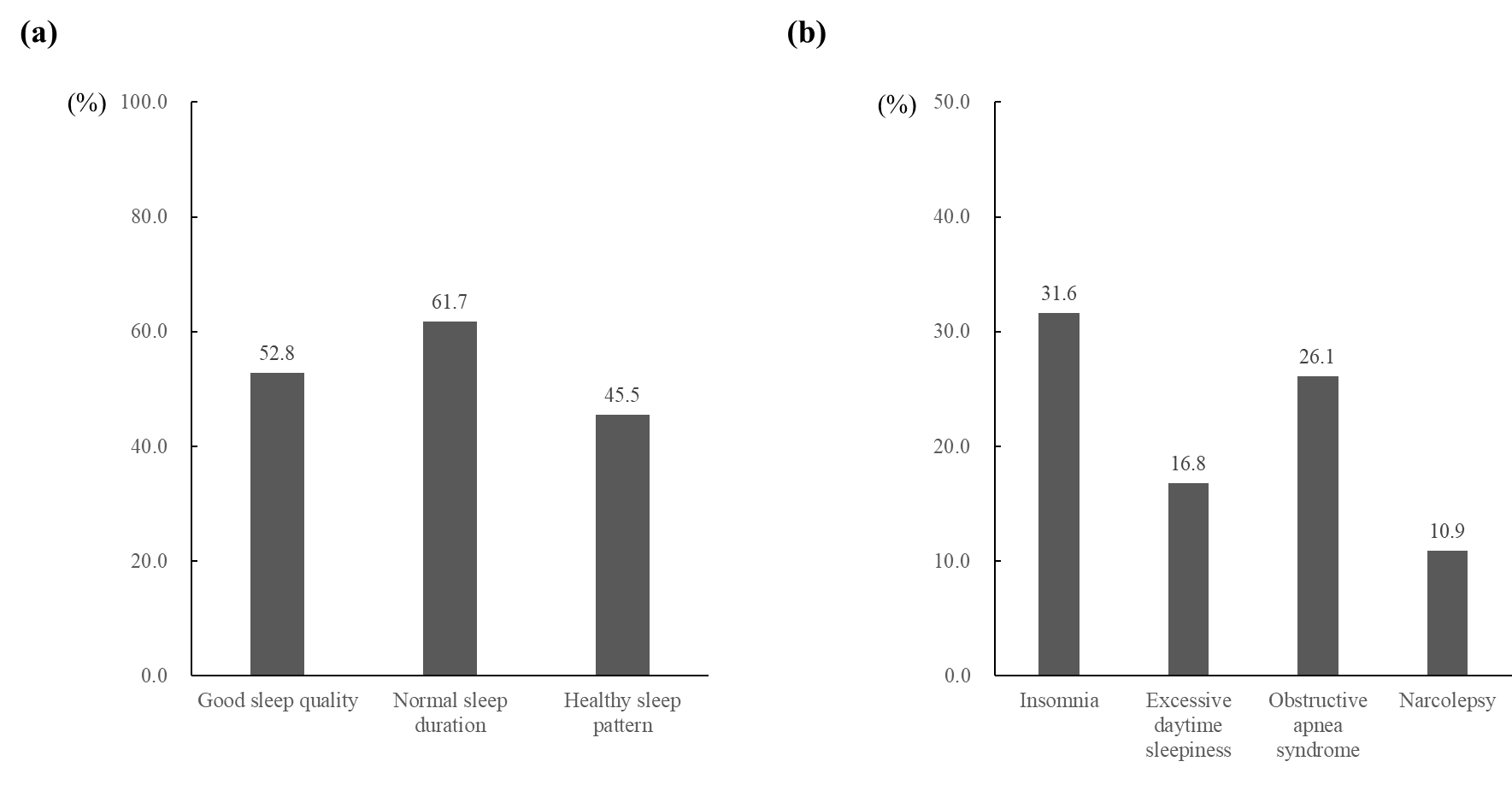


**Supplementary Table 1. Summary of the questionnaire related to this study.**

| **Questions** | **Types** | **Choices** |
| --- | --- | --- |
| **Part One: Demographic information** | | |
| Please fill in your age (years): | Blank | / |
| Please select your gender: | Single choice | Male/Female |
| Please fill in your permanent  geographical region: | Blank | / |
| Please select your ethnicity: | Single choice | Han/Others |
| Please select your highest level of  education: | Single choice | Elementary school or below/Junior high school/Senior high school or vocational school/Bachelor degree or college diploma/Master degree or above |
| Please select your monthly income (yuan): | Single choice | <2500/2500-4999/5000-9999/10000-19999/20000-34999/35000-50000/50000-100000/≥100000 |
| Please select your current marital  status: | Single choice | Married/Unmarried/Separate/Widow/Other_(Please specify) |
| Please select your current occupation: | Single choice | Civil servants/ Institutional staff/Enterprise or company employees/Private or individual workers/Freelancers (e.g., writers, artists, anchors, etc.)/Medical workers/  Service workers/Servicemen or military occupations/  Workers/Students/Farmers/Full-time caretaker of a family/Unemployed or jobless/Retired/Other_ (please specify) |
| Your current living area is： | Single choice | Urban/Rural |
| Are you living alone | Single choice | Yes/No |
| Do you have the habit of taking a nap: | Single choice | Never or almost never/Occasionally (1-3 times/month)/Sometimes (1-2 times/week)/ Often (3-5 times/week)/Almost every day |
| Do you have shift work in your current job? | Single choice | No, daytime work only/ Occasional shift work (1-3 times /month)/ Frequent shift work (≥1 time/week) |
| Have you been diagnosed by a doctor as (having) suffered from any of the following chronic disease? | Multiple choices | None [mutually exclusive] /Hypertension/Coronary heart disease/ Hyperlipidaemia or total cholesterol ≥ 6.5 mmol/L/Cerebrovascular disease or stroke/ Brain injury due to trauma/Migraine/ Diabetes mellitus/Chronic obstructive pulmonary disease/Asthma/Thyroid disease /Arthritis/Chronic pain/Malignant neoplasm (cancer)/  Parkinson's disease/Epilepsy/ Dementia/Mild cognitive impairment |
| Have you been diagnosed by a doctor as (having) suffered from any of the following mental illness? | Multiple choices | Depressive disorders/Bipolar disorders/ Anxious disorders/Obsessive-compulsive disorders/Schizophrenia/Other psychiatric disorders_ (please specify) |
| Have you ever been infected with COVID-19? | Single choice | Confirmed infection/ Asymptomatic infection/No |
| Have you ever been quarantined due to COVID-19 epidemics? | Single choice | Yes, being quarantined currently/ Yes, ever quarantined/No |
| **Part Two: Healthy lifestyles** | | |
| How often have you consumed the following foods in the past year: |  |  |
| Meat (Pork, Beef, Lamb & Poultry) | Single choice | Every day/4-6 days a week/1-3 days a week/ ≥1 time a month/ Never or almost never |
| Fish / Fish Products | Single choice | Every day/4-6 days a week/1-3 days a week/ ≥1 time a month/ Never or almost never |
| Fresh Eggs | Single choice | Every day/4-6 days a week/1-3 days a week/ ≥1 time a month/ Never or almost never |
| Fresh Vegetables | Single choice | Every day/4-6 days a week/1-3 days a week/ ≥1 time a month/ Never or almost never |
| Soya bean products | Single choice | Every day/4-6 days a week/1-3 days a week/ ≥1 time a month/ Never or almost never |
| Fresh Fruit | Single choice | Every day/4-6 days a week/1-3 days a week/ ≥1 time a month/ Never or almost never |
| Dairy products (milk, yoghurt) | Single choice | Every day/4-6 days a week/1-3 days a week/ ≥1 time a month/ Never or almost never |
| Sweets: e.g. desserts, ice creams | Single choice | Every day/4-6 days a week/1-3 days a week/ ≥1 time a month/ Never or almost never |
| Fried foods: e.g. fried chicken, crisps, etc. | Single choice | Every day/4-6 days a week/1-3 days a week/ ≥1 time a month/ Never or almost never |
| How often do you exercise daily? | Single choice | Never or almost never/1-3 times a month/1-2 times a week/3-5 times a week/ every day or almost every day |
| The intensity of your daily exercise is: | Single choice | Mild/Moderate/Vigorous |
| What is your average exercise duration each day? | Blank (number only) | _ Minutes |
| Are you a smoker (at least 1 cigarette per day for 6 months or more)? | Single choice | Never or occasionally/ Currently abstinent from smoking for at least 1 consecutive year (previous average of _ cigarettes/day for _ years)/ Yes (average_ cigarettes/day for _ years) |
| Do you have a habit of drinking alcohol? (more than 1 glass of beer or 1 shot of spirits of low alcoholic strength at least once a week for 6 months or more) | Single choice | Never or occasionally/ Currently abstinent from alcohol for at least 1 consecutive year (previous average of _ drinks/month, with less than one drink not counted, for a total of _ years)/ Yes (average _ times/month, less than one cup excluded, for _ years) |
| In the past year, how many hours per day, on average, did you spend sedentary time (i.e., sitting at work or for recreation)? | Blank (number only) | __ hours |
| Please fill in your height and weight | Blank (number only) | _ cm; _ kg |

**Supplementary Table 2. Logistic regression results of factors associated with sleep quality, normal sleep duration, and healthy sleep pattern.**

| **Variables** | **Good sleep quality** | | **Normal sleep duration** | | **Healthy sleep pattern** | |
| --- | --- | --- | --- | --- | --- | --- |
|  | **AOR (95% CI)** | **p value** | **AOR (95% CI)** | **p value** | **AOR (95% CI)** | **p value** |
| Average healthy lifestyle group (ref: unfavourable group) | 1.25 (1.18, 1.32) | <0.001 | 1.24 (1.18, 1.31) | <0.001 | 1.53 (1.45, 1.62) | <0.001 |
| Favourable healthy lifestyle group (ref: unfavourable group) | 1.56 (1.46, 1.68) | <0.001 | 1.60 (1.49, 1.72) | <0.001 | 2.15 (2.00, 2.31) | <0.001 |
| Age | 1.00 (1.00, 1.00) | 0.172 | 0.96 (0.96, 0.97) | <0.001 | 1.00 (0.99, 1.00) | 0.079 |
| Male (ref: female) | 1.37 (1.31, 1.43) | <0.001 | 1.02 (0.98, 1.07) | 0.348 | 1.00 (0.96, 1.05) | 0.999 |
| Han ethnicity (ref: others) | 1.06 (0.95, 1.19) | 0.293 | 1.02 (0.91, 1.14) | 0.731 | 1.08 (0.96, 1.21) | 0.207 |
| College or university (ref: high school or lower) | 0.92 (0.86, 0.97) | 0.003 | 0.94 (0.88, 0.99) | 0.023 | 0.85 (0.80, 0.90) | <0.001 |
| College or university (ref: postgraduate) | 1.04 (0.95, 1.14) | 0.372 | 0.92 (0.84, 1.01) | 0.067 | 0.82 (0.75, 0.90) | <0.001 |
| 5000-19999 yuan/month (ref: ≤4999 yuan/month) | 1.04 (0.99, 1.10) | 0.106 | 1.05 (0.99, 1.10) | 0.089 | 0.97 (0.92, 1.02) | 0.194 |
| ≥20000 yuan/month (ref: ≤4999 yuan/month) | 1.07 (1.00, 1.14) | 0.059 | 1.08 (1.01, 1.16) | 0.024 | 0.94 (0.88, 1.00) | 0.068 |
| Married (ref: unmarried) | 1.07 (1.00, 1.13) | 0.040 | 1.14 (1.07, 1.21) | <0.001 | 1.19 (1.12, 1.26) | <0.001 |
| Separate, widow, or others (ref: unmarried) | 0.84 (0.72, 0.98) | 0.022 | 0.86 (0.75, 0.99) | 0.042 | 0.86 (0.73, 1.00) | 0.043 |
| Manual work (ref: non-manual work) | 1.05 (0.99, 1.11) | 0.086 | 1.08 (1.02, 1.14) | 0.008 | 1.03 (0.98, 1.09) | 0.266 |
| Jobless (ref: non-manual work) | 1.01 (0.95, 1.08) | 0.781 | 1.09 (1.02, 1.16) | 0.010 | 0.90 (0.85, 0.96) | 0.002 |
| Urban area (ref: rural area) | 0.97 (0.90, 1.05) | 0.522 | 0.95 (0.87, 1.02) | 0.159 | 0.88 (0.81, 0.95) | 0.001 |
| Living alone (ref: no) | 0.93 (0.87, 0.99) | 0.017 | 1.05 (0.98, 1.11) | 0.142 | 0.91 (0.86, 0.97) | 0.005 |
| Napping less frequently (ref: no) | 0.70 (0.66, 0.74) | <0.001 | 0.95 (0.89, 1.00) | 0.058 | 0.84 (0.79, 0.89) | <0.001 |
| Napping frequently (ref: no) | 0.78 (0.74, 0.83) | <0.001 | 0.89 (0.85, 0.95) | <0.001 | 0.84 (0.79, 0.89) | <0.001 |
| Shiftwork (ref: no) | 0.66 (0.63, 0.69) | <0.001 | 0.69 (0.66, 0.73) | <0.001 | 0.59 (0.56, 0.63) | <0.001 |
| History of chronic disease (ref: no) | 0.49 (0.46, 0.52) | <0.001 | 0.70 (0.66, 0.74) | <0.001 | 0.41 (0.39, 0.44) | <0.001 |
| History of mental illness (ref: no) | 0.43 (0.36, 0.51) | <0.001 | 0.74 (0.64, 0.85) | <0.001 | 0.48 (0.40, 0.58) | <0.001 |
| Depression symptom (ref: no) | 0.40 (0.37, 0.43) | <0.001 | 0.69 (0.64, 0.74) | <0.001 | 0.41 (0.38, 0.44) | <0.001 |
| Anxiety symptom (ref: no) | 0.52 (0.48, 0.56) | <0.001 | 0.70 (0.65, 0.75) | <0.001 | 0.51 (0.47, 0.56) | <0.001 |
| Asymptomatic COVID-19 infection (ref: no) | 1.01 (0.85, 1.20) | 0.884 | 0.90 (0.77, 1.04) | 0.156 | 0.84 (0.70, 1.01) | 0.062 |
| Confirmed COVID-19 infection (ref: no) | 1.40 (1.15, 1.69) | 0.001 | 0.84 (0.71, 1.01) | 0.057 | 1.14 (0.94, 1.37) | 0.178 |
| Ever quarantined (ref: no) | 1.17 (0.90, 1.51) | 0.238 | 0.96 (0.76, 1.21) | 0.707 | 0.78 (0.60, 1.02) | 0.068 |
| Being quarantined currently (ref: no) | 0.72 (0.66, 0.79) | <0.001 | 0.88 (0.81, 0.96) | 0.003 | 0.70 (0.64, 0.77) | <0.001 |

Abbreviations: AOR: Adjusted Odds Ration, COVID-19: coronavirus disease 2019.

**Supplementary Table 3. Logistic regression results of factors associated with sleep disturbances.**

| **Variables** | **Insomnia** | | **Excessive daytime sleepiness** | | **Obstructive apnea syndrome** | | **Narcolepsy** | |
| --- | --- | --- | --- | --- | --- | --- | --- | --- |
|  | **AOR (95% CI)** | **p value** | **AOR (95% CI)** | **p value** | **AOR (95% CI)** | **p value** | **AOR (95% CI)** | **p value** |
| Average healthy lifestyle group (ref: unfavourable group) | 0.80 (0.75, 0.85) | <0.001 | 0.81 (0.75, 0.86) | <0.001 | 0.60 (0.56, 0.63) | <0.001 | 0.99 (0.91, 1.07) | 0.807 |
| Favourable healthy lifestyle group (ref: unfavourable group) | 0.66 (0.61, 0.71) | <0.001 | 0.66 (0.60, 0.73) | <0.001 | 0.40 (0.37, 0.43) | <0.001 | 0.92 (0.83, 1.03) | 0.159 |
| Age | 1.00 (1.00, 1.01) | 0.019 | 0.98 (0.98, 0.99) | <0.001 | 1.04 (1.03, 1.04) | <0.001 | 0.98 (0.98, 0.98) | <0.001 |
| Male (ref: female) | 0.83 (0.79, 0.87) | <0.001 | 1.00 (0.94, 1.06) | 0.969 | 3.93 (3.73, 4.14) | <0.001 | 1.11 (1.03, 1.19) | 0.004 |
| Han ethnicity (ref: others) | 0.92 (0.82, 1.04) | 0.195 | 1.07 (0.91, 1.25) | 0.406 | 0.91 (0.79, 1.04) | 0.152 | 0.93 (0.78, 1.11) | 0.440 |
| College or university (ref: high school or lower) | 1.20 (1.13, 1.28) | <0.001 | 1.04 (0.96, 1.12) | 0.336 | 0.84 (0.79, 0.90) | <0.001 | 0.78 (0.71, 0.85) | <0.001 |
| College or university (ref: postgraduate) | 1.14 (1.03, 1.26) | 0.009 | 1.06 (0.94, 1.20) | 0.354 | 0.78 (0.70, 0.86) | <0.001 | 0.69 (0.60, 0.80) | <0.001 |
| 5000-19999 yuan/month (ref: ≤4999 yuan/month) | 1.02 (0.96, 1.08) | 0.474 | 1.08 (1.01, 1.16) | 0.034 | 0.91 (0.86, 0.97) | 0.005 | 1.06 (0.98, 1.15) | 0.173 |
| ≥20000 yuan/month (ref: ≤4999 yuan/month) | 1.01 (0.94, 1.08) | 0.845 | 1.01 (1.00, 1.20) | 0.052 | 0.99 (0.91, 1.08) | 0.840 | 1.22 (1.09, 1.35) | <0.001 |
| Married (ref: unmarried) | 0.83 (0.78, 0.88) | <0.001 | 0.94 (0.86, 1.01) | 0.102 | 1.05 (0.97, 1.13) | 0.230 | 0.99 (0.91, 1.09) | 0.878 |
| Separate, widow, or others (ref: unmarried) | 1.09 (0.93, 1.27) | 0.270 | 0.97 (0.79, 1.18) | 0.728 | 0.92 (0.78, 1.09) | 0.331 | 0.93 (0.74, 1.17) | 0.542 |
| Manual work (ref: non-manual work) | 0.99 (0.93, 1.05) | 0.675 | 1.03 (0.96, 1.12) | 0.385 | 1.05 (0.98, 1.12) | 0.174 | 1.15 (1.06, 1.26) | 0.001 |
| Jobless (ref: non-manual work) | 1.07 (1.00, 1.14) | 0.051 | 1.04 (0.95, 1.13) | 0.420 | 1.15 (1.07, 1.24) | <0.001 | 1.05 (0.95, 1.15) | 0.377 |
| Urban area (ref: rural area) | 1.19 (1.09, 1.30) | <0.001 | 0.93 (0.84, 1.03) | 0.172 | 0.92 (0.84, 1.01) | 0.073 | 0.85 (0.76, 0.96) | 0.006 |
| Living alone (ref: no) | 1.08 (1.01, 1.15) | 0.028 | 0.97 (0.90, 1.05) | 0.461 | 0.96 (0.89, 1.03) | 0.233 | 0.94 (0.86, 1.03) | 0.197 |
| Napping less frequently (ref: no) | 1.34 (1.25, 1.43) | <0.001 | 1.26 (1.16, 1.37) | <0.001 | 1.05 (0.98, 1.12) | 0.183 | 1.81 (1.63, 2.01) | <0.001 |
| Napping frequently (ref: no) | 1.28 (1.20, 1.36) | <0.001 | 1.33 (1.22, 1.43) | <0.001 | 1.03 (0.96, 1.10) | 0.457 | 2.19 (1.98, 2.43) | <0.001 |
| Shiftwork (ref: no) | 1.65 (1.57, 1.74) | <0.001 | 1.37 (1.29, 1.46) | <0.001 | 1.27 (1.20, 1.35) | <0.001 | 1.52 (1.41, 1.63) | <0.001 |
| History of chronic disease (ref: no) | 2.10 (1.98, 2.23) | <0.001 | 1.70 (1.58, 1.82) | <0.001 | 2.86 (2.68, 3.04) | <0.001 | 2.07 (1.92, 2.24) | <0.001 |
| History of mental illness (ref: no) | 2.12 (1.82, 2.47) | <0.001 | 1.46 (1.25, 1.71) | <0.001 | 1.08 (0.93, 1.27) | 0.312 | 1.76 (1.49, 2.06) | <0.001 |
| Depression symptom (ref: no) | 2.58 (2.40, 2.78) | <0.001 | 2.60 (2.39, 2.83) | <0.001 | 1.13 (1.04, 1.24) | 0.006 | 1.42 (1.28, 1.58) | <0.001 |
| Anxiety symptom (ref: no) | 1.92 (1.77, 2.08) | <0.001 | 2.60 (2.38, 2.83) | <0.001 | 1.35 (1.23, 1.48) | <0.001 | 2.03 (1.83, 2.26) | <0.001 |
| Asymptomatic COVID-19 infection (ref: no) | 0.67 (0.57, 0.79) | <0.001 | 1.77 (1.50, 2.11) | <0.001 | 1.72 (1.46, 2.03) | <0.001 | 2.64 (2.23, 3.13) | <0.001 |
| Confirmed COVID-19 infection (ref: no) | 0.41 (0.33, 0.51) | <0.001 | 1.18 (0.95, 1.46) | 0.144 | 2.26 (1.86, 2.73) | <0.001 | 1.45 (1.15, 1.84) | 0.002 |
| Ever quarantined (ref: no) | 0.87 (0.66, 1.15) | 0.325 | 1.32 (1.00, 1.74) | 0.048 | 1.62 (1.25, 2.10) | <0.001 | 0.93 (0.69, 1.27) | 0.666 |
| Being quarantined currently (ref: no) | 1.38 (1.26, 1.51) | <0.001 | 1.31 (1.18, 1.46) | <0.001 | 1.01 (0.91, 1.11) | 0.855 | 1.11 (0.98, 1.26) | 0.090 |

Abbreviations: AOR: Adjusted Odds Ration, COVID-19: coronavirus disease 2019.

**Supplementary Table 4. Logistic regression results of each healthy lifestyle component associated with sleep quality, normal sleep duration, and healthy sleep pattern.**

| **Variables** | **Good sleep quality** | | **Normal sleep duration** | | **Healthy sleep pattern** | |
| --- | --- | --- | --- | --- | --- | --- |
|  | **AOR (95% CI)** | **p value** | **AOR (95% CI)** | **p value** | **AOR (95% CI)** | **p value** |
| Healthy diet (ref: no) | 1.17 (1.12, 1.22) | <0.001 | 1.27 (1.22, 1.33) | <0.001 | 1.31 (1.25, 1.36) | <0.001 |
| Regular exercise (ref: no) | 1.25 (1.19, 1.32) | <0.001 | 1.20 (1.14, 1.26) | <0.001 | 1.3 (1.24, 1.36) | <0.001 |
| Never smoking (ref: yes) | 1.18 (1.10, 1.27) | <0.001 | 1.17 (1.09, 1.26) | <0.001 | 1.41 (1.31, 1.52) | <0.001 |
| Never drinking alcohol (ref: yes) | 1.32 (1.20, 1.45) | <0.001 | 1.11 (1.02, 1.21) | 0.019 | 1.29 (1.17, 1.42) | <0.001 |
| Low sedentary behavior (ref: yes) | 1.19 (1.14, 1.25) | <0.001 | 1.11 (1.06, 1.16) | <0.001 | 1.32 (1.26, 1.38) | <0.001 |
| Normal weight (ref: no) | 0.97 (0.93, 1.02) | 0.215 | 1.08 (1.04, 1.13) | <0.001 | 1.13 (1.09, 1.18) | <0.001 |
| Age | 1.00 (1.00, 1.00) | 0.364 | 0.96 (0.96, 0.97) | <0.001 | 1.00 (0.99, 1.00) | 0.051 |
| Male (ref: female) | 1.38 (1.31, 1.45) | <0.001 | 1.03 (0.99, 1.09) | 0.173 | 1.03 (0.98, 1.08) | 0.291 |
| Han ethnicity (ref: others) | 1.07 (0.95, 1.20) | 0.275 | 1.02 (0.91, 1.14) | 0.781 | 1.07 (0.96, 1.21) | 0.232 |
| College or university (ref: high school or lower) | 0.92 (0.87, 0.98) | 0.005 | 0.93 (0.88, 0.98) | 0.009 | 0.85 (0.80, 0.90) | <0.001 |
| College or university (ref: postgraduate) | 1.04 (0.95, 1.14) | 0.419 | 0.90 (0.82, 0.98) | 0.020 | 0.81 (0.74, 0.89) | <0.001 |
| 5000-19999 yuan/month (ref: ≤4999 yuan/month) | 1.05 (0.99, 1.10) | 0.094 | 1.04 (0.99, 1.09) | 0.163 | 0.96 (0.92, 1.02) | 0.182 |
| ≥20000 yuan/month (ref: ≤4999 yuan/month) | 1.07 (1.00, 1.14) | 0.063 | 1.07 (1.00, 1.14) | 0.053 | 0.94 (0.87, 1.00) | 0.055 |
| Married (ref: unmarried) | 1.07 (1.01, 1.14) | 0.029 | 1.14 (1.07, 1.21) | <0.001 | 1.19 (1.11, 1.26) | <0.001 |
| Separate, widow, or others (ref: unmarried) | 0.85 (0.73, 0.99) | 0.034 | 0.87 (0.75, 1.00) | 0.057 | 0.87 (0.74, 1.01) | 0.063 |
| Manual work (ref: non-manual work) | 1.05 (0.99, 1.11) | 0.109 | 1.09 (1.03, 1.15) | 0.005 | 1.03 (0.97, 1.09) | 0.288 |
| Jobless (ref: non-manual work) | 0.99 (0.93, 1.06) | 0.783 | 1.08 (1.02, 1.15) | 0.015 | 0.89 (0.83, 0.95) | <0.001 |
| Urban area (ref: rural area) | 0.98 (0.90, 1.06) | 0.544 | 0.94 (0.87, 1.02) | 0.130 | 0.88 (0.81, 0.95) | 0.001 |
| Living alone (ref: no) | 0.92 (0.87, 0.98) | 0.011 | 1.04 (0.98, 1.10) | 0.227 | 0.91 (0.85, 0.97) | 0.003 |
| Napping less frequently (ref: no) | 0.70 (0.66, 0.74) | <0.001 | 0.94 (0.89, 1.00) | 0.036 | 0.83 (0.78, 0.88) | <0.001 |
| Napping frequently (ref: no) | 0.77 (0.73, 0.82) | <0.001 | 0.88 (0.83, 0.93) | <0.001 | 0.83 (0.78, 0.87) | <0.001 |
| Shiftwork (ref: no) | 0.66 (0.63, 0.70) | <0.001 | 0.70 (0.66, 0.73) | <0.001 | 0.60 (0.57, 0.63) | <0.001 |
| History of chronic disease (ref: no) | 0.48 (0.46, 0.51) | <0.001 | 0.70 (0.66, 0.74) | <0.001 | 0.41 (0.39, 0.44) | <0.001 |
| History of mental illness (ref: no) | 0.43 (0.36, 0.51) | <0.001 | 0.74 (0.64, 0.85) | <0.001 | 0.49 (0.40, 0.59) | <0.001 |
| Depression symptom (ref: no) | 0.40 (0.37, 0.44) | <0.001 | 0.70 (0.65, 0.75) | <0.001 | 0.41 (0.38, 0.45) | <0.001 |
| Anxiety symptom (ref: no) | 0.52 (0.48, 0.56) | <0.001 | 0.70 (0.65, 0.76) | <0.001 | 0.52 (0.47, 0.56) | <0.001 |
| Asymptomatic COVID-19 infection (ref: no) | 1.01 (0.85, 1.20) | 0.924 | 0.91 (0.78, 1.05) | 0.204 | 0.84 (0.70, 1.00) | 0.054 |
| Confirmed COVID-19 infection (ref: no) | 1.40 (1.15, 1.69) | 0.001 | 0.86 (0.72, 1.02) | 0.084 | 1.14 (0.95, 1.38) | 0.162 |
| Ever quarantined (ref: no) | 1.17 (0.91, 1.52) | 0.226 | 0.96 (0.76, 1.21) | 0.729 | 0.78 (0.60, 1.01) | 0.063 |
| Being quarantined currently (ref: no) | 0.72 (0.66, 0.79) | <0.001 | 0.88 (0.81, 0.96) | 0.002 | 0.70 (0.64, 0.77) | <0.001 |

Abbreviations: AOR: Adjusted Odds Ration, COVID-19: coronavirus disease 2019.

**Supplementary Table 5. Logistic regression results of each healthy lifestyle component associated with sleep disturbances.**

| **Variables** | **Insomnia** | | **Excessive daytime sleepiness** | | **Obstructive apnea syndrome** | | **Narcolepsy** | |
| --- | --- | --- | --- | --- | --- | --- | --- | --- |
|  | **AOR (95% CI)** | **p value** | **AOR (95% CI)** | **p value** | **AOR (95% CI)** | **p value** | **AOR (95% CI)** | **p value** |
| Healthy diet (ref: no) | 0.89 (0.85, 0.93) | <0.001 | 0.83 (0.78, 0.88) | <0.001 | 0.81 (0.77, 0.85) | <0.001 | 0.79 (0.74, 0.84) | <0.001 |
| Regular exercise (ref: no) | 0.83 (0.78, 0.87) | <0.001 | 0.89 (0.83, 0.96) | 0.001 | 0.85 (0.80, 0.90) | <0.001 | 1.14 (1.06, 1.23) | 0.001 |
| Never smoking (ref: yes) | 0.85 (0.78, 0.91) | <0.001 | 0.82 (0.75, 0.90) | <0.001 | 0.78 (0.72, 0.84) | <0.001 | 0.93 (0.83, 1.03) | 0.155 |
| Never drinking alcohol (ref: yes) | 0.74 (0.67, 0.81) | <0.001 | 0.91 (0.81, 1.02) | 0.099 | 0.88 (0.80, 0.96) | 0.006 | 0.88 (0.78, 1.00) | 0.057 |
| Low sedentary behavior (ref: yes) | 0.75 (0.71, 0.78) | <0.001 | 0.90 (0.84, 0.95) | <0.001 | 0.91 (0.86, 0.96) | <0.001 | 1.16 (1.08, 1.24) | <0.001 |
| Normal weight (ref: no) | 1.10 (1.05, 1.15) | <0.001 | 0.91 (0.86, 0.97) | 0.002 | 0.51 (0.48, 0.54) | <0.001 | 1.00 (0.94, 1.07) | 0.981 |
| Age | 1.00 (1.00, 1.01) | 0.011 | 0.98 (0.98, 0.99) | <0.001 | 1.04 (1.03, 1.04) | <0.001 | 0.98 (0.98, 0.98) | <0.001 |
| Male (ref: female) | 0.83 (0.79, 0.88) | <0.001 | 0.97 (0.91, 1.04) | 0.410 | 3.75 (3.54, 3.97) | <0.001 | 1.04 (0.96, 1.12) | 0.362 |
| Han ethnicity (ref: others) | 0.92 (0.81, 1.04) | 0.172 | 1.07 (0.92, 1.26) | 0.372 | 0.92 (0.80, 1.05) | 0.229 | 0.95 (0.80, 1.13) | 0.553 |
| College or university (ref: high school or lower) | 1.17 (1.10, 1.25) | <0.001 | 1.05 (0.97, 1.13) | 0.243 | 0.87 (0.81, 0.93) | <0.001 | 0.80 (0.74, 0.87) | <0.001 |
| College or university (ref: postgraduate) | 1.12 (1.01, 1.23) | 0.031 | 1.08 (0.95, 1.22) | 0.228 | 0.81 (0.73, 0.90) | <0.001 | 0.72 (0.63, 0.84) | <0.001 |
| 5000-19999 yuan/month (ref: ≤4999 yuan/month) | 1.01 (0.96, 1.07) | 0.712 | 1.08 (1.01, 1.16) | 0.027 | 0.93 (0.87, 0.99) | 0.021 | 1.06 (0.98, 1.16) | 0.133 |
| ≥20000 yuan/month (ref: ≤4999 yuan/month) | 1.00 (0.93, 1.07) | 0.951 | 1.10 (1.00, 1.21) | 0.043 | 1.01 (0.93, 1.09) | 0.861 | 1.22 (1.09, 1.35) | <0.001 |
| Married (ref: unmarried) | 0.83 (0.78, 0.89) | <0.001 | 0.94 (0.86, 1.01) | 0.099 | 1.04 (0.97, 1.12) | 0.295 | 0.99 (0.90, 1.08) | 0.763 |
| Separate, widow, or others (ref: unmarried) | 1.09 (0.93, 1.27) | 0.291 | 0.96 (0.78, 1.17) | 0.655 | 0.92 (0.78, 1.10) | 0.362 | 0.91 (0.72, 1.15) | 0.450 |
| Manual work (ref: non-manual work) | 1.00 (0.94, 1.06) | 1.00 | 1.03 (0.95, 1.11) | 0.454 | 1.03 (0.96, 1.10) | 0.420 | 1.13 (1.04, 1.23) | 0.005 |
| Jobless (ref: non-manual work) | 1.10 (1.03, 1.18) | 0.005 | 1.04 (0.96, 1.13) | 0.360 | 1.13 (1.05, 1.23) | 0.001 | 1.03 (0.93, 1.13) | 0.615 |
| Urban area (ref: rural area) | 1.18 (1.08, 1.29) | <0.001 | 0.93 (0.84, 1.03) | 0.173 | 0.93 (0.85, 1.02) | 0.107 | 0.86 (0.77, 0.96) | 0.010 |
| Living alone (ref: no) | 1.08 (1.01, 1.15) | 0.025 | 0.98 (0.90, 1.06) | 0.567 | 0.96 (0.89, 1.03) | 0.240 | 0.96 (0.87, 1.05) | 0.343 |
| Napping less frequently (ref: no) | 1.35 (1.27, 1.44) | <0.001 | 1.27 (1.17, 1.37) | <0.001 | 1.04 (0.97, 1.12) | 0.258 | 1.81 (1.63, 2.01) | <0.001 |
| Napping frequently (ref: no) | 1.29 (1.21, 1.37) | <0.001 | 1.34 (1.24, 1.45) | <0.001 | 1.02 (0.95, 1.09) | 0.545 | 2.21 (2.00, 2.45) | <0.001 |
| Shiftwork (ref: no) | 1.66 (1.58, 1.75) | <0.001 | 1.36 (1.28, 1.45) | <0.001 | 1.26 (1.19, 1.34) | <0.001 | 1.49 (1.38, 1.60) | <0.001 |
| History of chronic disease (ref: no) | 2.11 (1.99, 2.24) | <0.001 | 1.69 (1.58, 1.82) | <0.001 | 2.82 (2.65, 3.00) | <0.001 | 2.06 (1.91, 2.23) | <0.001 |
| History of mental illness (ref: no) | 2.11 (1.81, 2.46) | <0.001 | 1.45 (1.24, 1.70) | <0.001 | 1.09 (0.93, 1.28) | 0.268 | 1.74 (1.48, 2.04) | <0.001 |
| Depression symptom (ref: no) | 2.55 (2.37, 2.75) | <0.001 | 2.59 (2.38, 2.81) | <0.001 | 1.15 (1.05, 1.25) | 0.003 | 1.42 (1.28, 1.58) | <0.001 |
| Anxiety symptom (ref: no) | 1.92 (1.78, 2.08) | <0.001 | 2.59 (2.38, 2.83) | <0.001 | 1.36 (1.24, 1.50) | <0.001 | 2.03 (1.82, 2.25) | <0.001 |
| Asymptomatic COVID-19 infection (ref: no) | 0.68 (0.57, 0.80) | <0.001 | 1.76 (1.49, 2.09) | <0.001 | 1.73 (1.47, 2.05) | <0.001 | 2.58 (2.18, 3.05) | <0.001 |
| Confirmed COVID-19 infection (ref: no) | 0.41 (0.33, 0.51) | <0.001 | 1.16 (0.94, 1.45) | 0.171 | 2.24 (1.85, 2.72) | <0.001 | 1.41 (1.12, 1.78) | 0.004 |
| Ever quarantined (ref: no) | 0.87 (0.66, 1.15) | 0.324 | 1.32 (1.00, 1.74) | 0.047 | 1.64 (1.27, 2.13) | <0.001 | 0.92 (0.68, 1.26) | 0.607 |
| Being quarantined currently (ref: no) | 1.37 (1.25, 1.50) | <0.001 | 1.31 (1.18, 1.46) | <0.001 | 1.02 (0.92, 1.12) | 0.757 | 1.11 (0.98, 1.26) | 0.089 |

Abbreviations: AOR: Adjusted Odds Ration, COVID-19: coronavirus disease 2019.
